# Supplementary material for: Clinical learning environments and experiences of nursing students in West Bank Universities: A mixed-methods study
Source: PLoS One. 2025 Aug 26;20(8):e0327506. doi: 10.1371/journal.pone.0327506 (PMC12380295; doi:10.1371/journal.pone.0327506)
Supplement: S1 File — (DOCX) [file pone.0327506.s001.docx]

**Supplementary File 1: Checklist of Mixed Methods Elements**

**Clinical Learning Environments and Experiences of Nursing Students in West Bank Universities: A Mixed-Methods Study**

**Based on O'Cathain et al. (2008) Quality Framework for Mixed Methods Studies**

**A. RATIONALE AND JUSTIFICATION**

| **Element** | **Present** | **Description** |
| --- | --- | --- |
| **Rationale for mixed methods approach** | ✓ | Clear justification provided for using mixed methods to examine both quantitative relationships and qualitative experiences in clinical learning environments |
| **Justification for specific mixed methods design** | ✓ | Convergent design selected to allow simultaneous collection and integration of quantitative and qualitative data |
| **Added value of mixed methods over single method** | ✓ | Demonstrates how quantitative data alone would miss contextual barriers and facilitators, while qualitative data alone would lack generalizability |

**B. DESIGN AND IMPLEMENTATION**

| **Element** | **Present** | **Description** |
| --- | --- | --- |
| **Mixed methods design clearly stated** | ✓ | Convergent mixed methods design explicitly identified |
| **Timing of data collection** | ✓ | Concurrent data collection from September to December 2023 |
| **Priority/emphasis given to each strand** | ✓ | Equal emphasis (QUAN + QUAL) with quantitative component (n=306) and qualitative component (n=14) |
| **Sampling strategy for each strand** | ✓ | Convenience sampling for quantitative; purposive sampling for qualitative |
| **Data collection procedures** | ✓ | Detailed procedures for both survey administration and semi-structured interviews |

**C. ANALYTICAL APPROACH**

| **Element** | **Present** | **Description** |
| --- | --- | --- |
| **Separate analysis of each strand** | ✓ | Quantitative: SPSS analysis including regression, ANOVA, correlations; Qualitative: Inductive content analysis using NVivo |
| **Integration method specified** | ✓ | Joint display approach for integration at interpretation level |
| **Integration timing** | ✓ | Integration occurred during interpretation phase after separate analyses |
| **Triangulation approach** | ✓ | Convergent, complementary, and contrasting findings identified |

**D. QUALITY AND RIGOR**

| **Element** | **Present** | **Description** |
| --- | --- | --- |
| **Validity/trustworthiness for quantitative strand** | ✓ | Validated Arabic CLES+T scale (α=0.984); assumption testing for parametric tests |
| **Validity/trustworthiness for qualitative strand** | ✓ | Inter-coder reliability (κ=0.85); member checking with 5 participants; dual coding process |
| **Integration validity** | ✓ | Joint display table showing convergent, complementary, and contrasting findings |
| **Reproducibility** | ✓ | Detailed methodology allows replication; instruments and procedures clearly described |

**E. REPORTING AND PRESENTATION**

| **Element** | **Present** | **Description** |
| --- | --- | --- |
| **Clear presentation of quantitative results** | ✓ | Tables, statistical analyses, and effect sizes reported |
| **Clear presentation of qualitative results** | ✓ | Themes presented with supporting quotes and participant demographics |
| **Integration results clearly presented** | ✓ | Table 3 provides clear integration of quantitative and qualitative findings |
| **Visual displays used effectively** | ✓ | Tables for quantitative data and joint display for integration |
| **Limitations addressed for both strands** | ✓ | Cross-sectional design, self-report bias, sampling limitations discussed |

**F. ETHICAL CONSIDERATIONS**

| **Element** | **Present** | **Description** |
| --- | --- | --- |
| **Ethical approval obtained** | ✓ | Arab American University-Palestine IRB approval (No: AAUP/IRB/2023-012) |
| **Informed consent for both strands** | ✓ | Written informed consent for survey and interviews |
| **Confidentiality measures** | ✓ | Data security measures including encryption and locked storage |
| **Voluntary participation** | ✓ | No academic penalty for withdrawal; independent data collection |

**G. CONTEXTUAL CONSIDERATIONS**

| **Element** | **Present** | **Description** |
| --- | --- | --- |
| **Cultural adaptation** | ✓ | CLES+T scale culturally adapted for Palestinian context |
| **Conflict-sensitive approach** | ✓ | Consideration of movement restrictions and security concerns |
| **Local expertise involvement** | ✓ | Expert panel for cultural adaptation; local research team |
| **Practical implications for context** | ✓ | Recommendations specific to conflict-affected educational settings |

**H. INNOVATION AND CONTRIBUTION**

| **Element** | **Present** | **Description** |
| --- | --- | --- |
| **Novel application of mixed methods** | ✓ | First mixed methods study of clinical learning environments in Palestinian context |
| **Methodological innovation** | ✓ | Adaptation of validated instrument for conflict-affected setting |
| **Theoretical contribution** | ✓ | Application of Dunn and Burnett's framework in conflict context |
| **Practical contribution** | ✓ | Evidence-based recommendations for similar contexts globally |

**OVERALL ASSESSMENT**

**Strengths:**

- Comprehensive mixed methods design with clear rationale
- Rigorous methodology with appropriate sample sizes
- Effective integration of quantitative and qualitative findings
- Strong cultural adaptation and contextual sensitivity
- Clear practical implications

**Areas for Enhancement:**

- Longitudinal design could strengthen causal inferences
- Broader geographic representation beyond West Bank
- More detailed explanation of integration challenges

**Quality Rating: EXCELLENT**

This mixed methods study demonstrates high methodological rigor, appropriate integration techniques, and meaningful contribution to nursing education research in conflict-affected settings.
