# Supplementary material for: Clinical learning environments and experiences of nursing students in West Bank Universities: A mixed-methods study
Source: PLoS One. 2025 Aug 26;20(8):e0327506. doi: 10.1371/journal.pone.0327506 (PMC12380295; doi:10.1371/journal.pone.0327506)
Supplement: S2 File — (DOCX) [file pone.0327506.s002.docx]

**Supplementary File 2: Interview Guide for Qualitative Data Collection**

**Clinical Learning Environments and Experiences of Nursing Students in West Bank Universities: A Mixed-Methods Study**

**Semi-Structured Interview Guide for Fourth-Year Nursing Students**

**PRE-INTERVIEW SETUP**

**Duration:** 45-60 minutes
**Setting:** Private room at participant's university
**Recording:** Audio recording with participant consent
**Materials:** Interview guide, audio recorder, field notes sheet, consent form

**Introduction Script:** "Thank you for agreeing to participate in this interview. We are exploring nursing students' experiences in clinical learning environments across West Bank universities. Your insights will help us understand what facilitates or hinders clinical learning in our context. This interview will last about 45-60 minutes, and with your permission, I will audio-record our conversation. All information you share will be kept confidential and your identity will remain anonymous. You can stop the interview at any time or skip any questions you don't wish to answer. Do you have any questions before we begin?"

**SECTION A: BACKGROUND AND CLINICAL EXPERIENCE**

**Opening Questions:**

1. **Clinical Background**
   - Can you tell me about your clinical rotations so far? Which departments/wards have you been placed in?
   - How many different clinical sites have you experienced during your studies?
   - Which clinical rotation has been most meaningful to you and why?
2. **Current Clinical Placement**
   - Describe your current clinical placement. What type of facility is it? (governmental, private, UNRWA)
   - What is a typical day like during your clinical rotation?
   - How many hours per week do you spend in clinical practice?

**SECTION B: PERCEPTIONS OF CLINICAL LEARNING ENVIRONMENT**

**Core Questions:**

1. **Overall Clinical Experience**
   - How would you describe your overall clinical learning experience?
   - What does an ideal clinical learning environment look like to you?
   - How does your actual experience compare to your expectations when you started nursing school?
2. **Pedagogical Atmosphere**
   - How would you describe the learning atmosphere in your clinical placements?
   - Tell me about the relationship between students and staff in clinical settings.
   - How do you feel when you're in the clinical environment? (comfortable, anxious, supported, etc.)
   - Can you give me an example of a time when you felt particularly supported or unsupported in clinical practice?
3. **Supervision and Mentorship**
   - Tell me about your clinical instructors/supervisors. What makes a good clinical instructor?
   - How do your clinical instructors support your learning?
   - Describe the feedback you receive during clinical practice.
   - How available are your instructors when you need help or guidance?
   - Can you share an example of particularly effective or ineffective supervision?
4. **Ward Management and Leadership**
   - How do ward managers and charge nurses interact with students?
   - Do you feel welcomed and included in the ward team?
   - How do senior staff members contribute to your learning?
   - Tell me about any conflicts or challenges you've experienced with ward leadership.

**SECTION C: FACILITATORS OF CLINICAL LEARNING**

**Facilitating Factors:**

1. **Positive Learning Experiences**
   - What aspects of your clinical placements have been most helpful for your learning?
   - Can you describe a clinical experience where you felt you learned the most?
   - What makes certain clinical sites better for learning than others?
   - How do supportive relationships with staff impact your learning?
2. **Effective Teaching Strategies**
   - What teaching methods in clinical settings work best for you?
   - How do you prefer to receive feedback on your clinical performance?
   - What role does hands-on practice play in your learning?
   - How important is it to have variety in your clinical experiences?
3. **Institutional Support**
   - How does your university prepare you for clinical practice?
   - What support do you receive from your nursing school during clinical rotations?
   - How well do theory and practice connect in your experience?

**SECTION D: BARRIERS TO CLINICAL LEARNING**

**Challenging Factors:**

1. **Resource and Structural Barriers**
   - What challenges do you face in accessing clinical learning opportunities?
   - How do resource limitations affect your clinical education?
   - Tell me about any times when you couldn't practice skills due to lack of equipment or supplies.
   - How does patient load affect your learning opportunities?
2. **Contextual and Political Barriers**
   - How do movement restrictions and checkpoints affect your clinical education?
   - Can you describe a situation where political or security issues impacted your clinical learning?
   - How do you cope with the stress of navigating these challenges?
   - What happens when you're delayed or cannot reach clinical sites?
3. **Educational and Professional Barriers**
   - What gaps do you see between classroom learning and clinical practice?
   - How do you handle situations where you feel unprepared for clinical tasks?
   - What non-educational tasks are you asked to do that might interfere with learning?
   - How do you deal with conflicts between different expectations from instructors and staff?

**SECTION E: SPECIFIC CLINICAL CONTEXTS**

**Contextual Variations:**

1. **Comparison Across Settings**
   - How do your experiences differ between governmental and private hospitals?
   - What differences have you noticed between different clinical departments?
   - How do urban versus rural clinical sites compare?
   - What makes UNRWA clinics different from hospital settings?
2. **Patient Interactions**
   - How do patients and families respond to having students involved in their care?
   - Can you describe a meaningful patient interaction that enhanced your learning?
   - How do cultural factors influence your clinical practice?
   - What challenges do you face when working with different patient populations?

**SECTION F: COPING STRATEGIES AND ADAPTATION**

**Adaptive Responses:**

1. **Personal Coping Mechanisms**
   - How do you manage stress during clinical rotations?
   - What strategies do you use to maximize your learning despite challenges?
   - How do you maintain motivation when facing difficult situations?
   - What support do you seek from peers, family, or instructors?
2. **Peer Support and Learning**
   - How do you and your classmates support each other during clinical practice?
   - What do you learn from observing and working with other students?
   - How do you handle competition or conflicts with other students?

**SECTION G: SUGGESTIONS FOR IMPROVEMENT**

**Recommendations:**

1. **Immediate Improvements**
   - What changes would most improve your clinical learning experience?
   - How could clinical instructors better support student learning?
   - What would make clinical sites more conducive to learning?
   - How could universities better prepare students for clinical practice?
2. **System-Level Changes**
   - What changes would you recommend to nursing education policy?
   - How could clinical partnerships between universities and hospitals be improved?
   - What role should simulation and technology play in clinical education?
   - How could the nursing curriculum be adapted to better fit the Palestinian context?
3. **Long-term Vision**
   - What would ideal clinical education look like in Palestine?
   - How could clinical learning be made more resilient to political and economic challenges?
   - What innovations or alternatives could enhance clinical education?

**SECTION H: CLOSING QUESTIONS**

**Wrap-up:**

1. **Future Perspectives**
   - How has your clinical experience shaped your view of nursing as a profession?
   - What are your career plans after graduation?
   - How confident do you feel about entering the workforce based on your clinical training?
2. **Final Reflections**
   - Is there anything important about your clinical learning experience that we haven't discussed?
   - What advice would you give to nursing students just starting their clinical rotations?
   - What message would you want to share with nursing educators and policymakers?

**CLOSING SCRIPT**

"Thank you so much for sharing your experiences with me. Your insights are invaluable for understanding clinical learning in our context. As mentioned, all information will be kept confidential, and we'll share the study findings with participating institutions. If you think of anything else you'd like to add, please feel free to contact me. Do you have any questions about the study or what happens next?"

**FIELD NOTES TEMPLATE**

**Post-Interview Notes:**

- **Date/Time:** ___________
- **Participant Code:** ___________
- **Location:** ___________
- **Interview Duration:** ___________
- **Overall Impression:** ___________
- **Key Themes Observed:** ___________
- **Non-verbal Cues:** ___________
- **Contextual Factors:** ___________
- **Follow-up Needed:** ___________

**PROBING QUESTIONS (USE AS NEEDED)**

- "Can you tell me more about that?"
- "What did that mean to you?"
- "How did that make you feel?"
- "Can you give me a specific example?"
- "What was that experience like?"
- "How did you handle that situation?"
- "What would you have preferred to happen?"
- "Is there anything else you'd like to add about that?"
