# Supplementary material for: Clinical learning environments and experiences of nursing students in West Bank Universities: A mixed-methods study
Source: PLoS One. 2025 Aug 26;20(8):e0327506. doi: 10.1371/journal.pone.0327506 (PMC12380295; doi:10.1371/journal.pone.0327506)
Supplement: S3 File — (DOCX) [file pone.0327506.s003.docx]

**Supplementary File 3: Demographic Questionnaire Details**

**Clinical Learning Environments and Experiences of Nursing Students in West Bank Universities: A Mixed-Methods Study**

**Demographic and Background Information Questionnaire**

**INSTRUCTIONS FOR PARTICIPANTS**

**Arabic Instructions:** يرجى ملء هذا الاستبيان بعناية. جميع المعلومات ستبقى سرية وستستخدم لأغراض البحث فقط. لا تكتب اسمك أو أي معلومات شخصية تحدد هويتك.

**English Instructions:** Please complete this questionnaire carefully. All information will remain confidential and will be used for research purposes only. Do not write your name or any personal identifying information.

**SECTION A: PERSONAL DEMOGRAPHICS**

**A1. Age**

- What is your age in years? _______ years
- Age categories for analysis:
  - 19-20 years
  - 21-22 years
  - 23-24 years
  - 25-26 years

**A2. Gender**

- Male (ذكر)
- Female (أنثى)

**A3. Marital Status**

- Single (أعزب/عزباء)
- Married (متزوج/متزوجة)
- Divorced (مطلق/مطلقة)
- Widowed (أرمل/أرملة)

**A4. Place of Residence**

- Nablus (نابلس)
- Ramallah (رام الله)
- Jenin (جنين)
- Tulkarm (طولكرم)
- Qalqilya (قلقيلية)
- Salfit (سلفيت)
- Bethlehem (بيت لحم)
- Hebron (الخليل)
- Jericho (أريحا)
- Other: _______________

**A5. Monthly Family Income (Optional)**

- Less than 1,000 NIS
- 1,000-2,000 NIS
- 2,001-3,000 NIS
- 3,001-4,000 NIS
- 4,001-5,000 NIS
- More than 5,000 NIS
- Prefer not to answer

**SECTION B: ACADEMIC INFORMATION**

**B1. Current Academic Year**

- Second year (السنة الثانية)
- Third year (السنة الثالثة)
- Fourth year (السنة الرابعة)

**B2. University Type and Name**

- Governmental University: _______________
- Public University: _______________
- Private University: _______________

**B3. Nursing Program Details**

- Program type: [ ] Bachelor of Science in Nursing (BSN)
- Expected graduation year: _______
- Current GPA (optional): _______

**B4. Previous Healthcare Experience**

- No previous healthcare experience
- Volunteer work in healthcare
- Paid work in healthcare
- Family member in healthcare profession
- Other: _______________

**B5. Motivation for Choosing Nursing**

- Personal interest in healthcare
- Job security
- Family influence
- Limited other options
- Desire to help others
- Financial reasons
- Other: _______________

**SECTION C: CLINICAL TRAINING INFORMATION**

**C1. Total Number of Clinical Rotations Completed**

- 1-2 rotations
- 3-4 rotations
- 5-6 rotations
- 7-8 rotations
- More than 8 rotations

**C2. Current Clinical Training Site**

- Governmental hospital
- Private hospital
- UNRWA clinic
- Primary healthcare center
- Other: _______________

**C3. Current Clinical Department/Ward**

- Medical ward (الطب الباطني)
- Surgical ward (الجراحة)
- Intensive Care Unit (العناية المركزة)
- Emergency Department (الطوارئ)
- Pediatrics (طب الأطفال)
- Obstetrics/Gynecology (التوليد وأمراض النساء)
- Orthopedics (العظام)
- Psychiatry/Mental Health (الطب النفسي)
- Community Health (صحة المجتمع)
- Operating Room (غرفة العمليات)
- Oncology (الأورام)
- Other: _______________

**C4. Clinical Training Schedule**

- Hours per week in clinical practice: _______ hours
- Number of days per week: _______ days
- Duration of current rotation: _______ weeks

**C5. Clinical Sites Experienced** *(Check all that apply)*

- An-Najah National University Hospital
- Rafidia Hospital
- Arab Specialized Hospital
- Al-Watani Hospital
- Thabet Thabet Hospital
- Palestine Medical Complex
- UNRWA clinics
- Other: _______________

**SECTION D: CLINICAL LEARNING CONTEXT**

**D1. Clinical Instructor Information**

- Number of clinical instructors you've worked with: _______
- Primary clinical instructor's experience:
  - Less than 2 years
  - 2-5 years
  - 6-10 years
  - More than 10 years
  - Don't know

**D2. Clinical Group Size**

- Typical number of students in your clinical group: _______
- Do you feel this group size is:
  - Too small
  - Appropriate
  - Too large

**D3. Patient-to-Student Ratio**

- On average, how many students share one patient during clinical practice?
  - 1 student per patient
  - 2-3 students per patient
  - 4-6 students per patient
  - 7-10 students per patient
  - More than 10 students per patient

**D4. Clinical Learning Resources** *(Check all that apply)*

- Adequate medical equipment
- Sufficient patient cases
- Access to patient records
- Simulation facilities
- Skills laboratory
- Computer/internet access
- Medical library
- Other: _______________

**SECTION E: CHALLENGES AND BARRIERS**

**E1. Transportation to Clinical Sites**

- How do you usually travel to clinical sites?
  - University transportation
  - Private car
  - Public transportation
  - Walking
  - Other: _______________

**E2. Checkpoint/Movement Restrictions**

- How often do checkpoints/movement restrictions affect your clinical attendance?
  - Never
  - Rarely (once per semester)
  - Sometimes (once per month)
  - Often (once per week)
  - Very often (more than once per week)

**E3. Average Time Lost Due to Checkpoints**

- Per clinical day: _______ minutes
- Has this ever caused you to miss clinical sessions?
  - Yes [ ] No
- If yes, how many times this semester? _______

**E4. Financial Challenges**

- Do you face financial difficulties that affect your clinical education?
  - Yes [ ] No
- If yes, specify:
  - Transportation costs
  - Uniform/equipment costs
  - Meal costs during long clinical days
  - Other: _______________

**SECTION F: TECHNOLOGY AND INNOVATION**

**F1. Use of Technology in Clinical Learning**

- Mobile apps for medical references
- Electronic health records
- Simulation software
- Online clinical resources
- Video conferencing for remote learning
- None of the above

**F2. Preferred Learning Methods** *(Rank from 1-5, where 1 is most preferred)*

- Direct patient care: _____
- Simulation exercises: _____
- Case study discussions: _____
- Observing procedures: _____
- Classroom integration: _____

**SECTION G: SUPPORT SYSTEMS**

**G1. Academic Support**

- Who do you turn to for academic support during clinical rotations? *(Check all that apply)*
- Clinical instructors
- Nursing faculty
- Fellow students
- Ward staff
- Family members
- Other: _______________

**G2. Emotional Support**

- How do you cope with stress during clinical rotations? *(Check all that apply)*
- Talk to friends/family
- Seek counseling services
- Religious/spiritual practices
- Physical exercise
- Recreational activities
- Other: _______________

**G3. Professional Development**

- Are you involved in any professional nursing organizations?
  - Yes [ ] No
- If yes, which ones? _______________

**SECTION H: FUTURE PLANS**

**H1. Career Intentions**

- After graduation, do you plan to:
  - Work in a Palestinian hospital
  - Work in a clinic/primary care
  - Continue to graduate school
  - Work abroad
  - Leave the nursing profession
  - Undecided

**H2. Specialty Interest**

- Which nursing specialty interests you most?
  - Medical-surgical nursing
  - Critical care nursing
  - Pediatric nursing
  - Obstetric nursing
  - Psychiatric nursing
  - Community health nursing
  - Emergency nursing
  - Other: _______________

**H3. Confidence Level**

- How confident do you feel about entering the nursing workforce?
  - Very confident
  - Somewhat confident
  - Neutral
  - Somewhat unconfident
  - Very unconfident

**SECTION I: ADDITIONAL INFORMATION**

**I1. Special Circumstances**

- Are there any special circumstances that affect your clinical learning?
  - Disability/health condition
  - Childcare responsibilities
  - Work obligations
  - Family responsibilities
  - Other: _______________

**I2. Additional Comments**

- Is there anything else you would like to share about your clinical learning experience?

**CODING INFORMATION (FOR RESEARCH USE ONLY)**

**Participant Code:** _______________ **Data Collection Date:** _______________ **University Site:** _______________ **Questionnaire Version:** V1.0 **Completed by:** _______________ **Quality Check:** _______________

**THANK YOU**

شكراً لك على مشاركتك في هذه الدراسة. مساهمتك ستساعد في تحسين التعليم التمريضي في الضفة الغربية.

Thank you for your participation in this study. Your contribution will help improve nursing education in the West Bank.

**For questions or concerns, contact:** Dr. Ibrahim Aqtam Email: [ibrahim.aqtam@nu-vte.edu.ps](mailto:ibrahim.aqtam@nu-vte.edu.ps) Phone: +972 597939465
