# Supplementary material for: Clinical learning environments and experiences of nursing students in West Bank Universities: A mixed-methods study
Source: PLoS One. 2025 Aug 26;20(8):e0327506. doi: 10.1371/journal.pone.0327506 (PMC12380295; doi:10.1371/journal.pone.0327506)
Supplement: S4 File — (DOCX) [file pone.0327506.s004.docx]

**Supplementary File 4: Additional Statistical Tables and Figures**

**Clinical Learning Environments and Experiences of Nursing Students in West Bank Universities: A Mixed-Methods Study**

**Table S1. Detailed Participant Characteristics by University Type**

| **Characteristic** | **Private Universities** | **Public Universities** | **Governmental Universities** | **Total** | **p-value** |
| --- | --- | --- | --- | --- | --- |
| **Total Participants** | 182 (59.5%) | 107 (34.9%) | 17 (5.6%) | 306 (100%) | - |
| **Gender** |  |  |  |  |  |
| Male | 58 (31.9%) | 32 (29.9%) | 7 (41.2%) | 97 (31.7%) | 0.627 |
| Female | 124 (68.1%) | 75 (70.1%) | 10 (58.8%) | 209 (68.3%) |  |
| **Age (years)** |  |  |  |  |  |
| Mean ± SD | 21.3 ± 1.7 | 21.6 ± 1.9 | 21.2 ± 1.8 | 21.4 ± 1.8 | 0.448 |
| Range | 19-26 | 19-26 | 19-25 | 19-26 |  |
| **Academic Year** |  |  |  |  |  |
| Second year | 44 (24.2%) | 26 (24.3%) | 5 (29.4%) | 75 (24.5%) | 0.892 |
| Third year | 61 (33.5%) | 34 (31.8%) | 5 (29.4%) | 100 (32.7%) |  |
| Fourth year | 77 (42.3%) | 47 (43.9%) | 7 (41.2%) | 131 (42.8%) |  |
| **Marital Status** |  |  |  |  |  |
| Single | 168 (92.3%) | 96 (89.7%) | 16 (94.1%) | 280 (91.5%) | 0.609 |
| Married | 14 (7.7%) | 11 (10.3%) | 1 (5.9%) | 26 (8.5%) |  |
| **Place of Residence** |  |  |  |  |  |
| Nablus | 89 (48.9%) | 52 (48.6%) | 8 (47.1%) | 149 (48.7%) | 0.234 |
| Ramallah | 31 (17.0%) | 23 (21.5%) | 3 (17.6%) | 57 (18.6%) |  |
| Jenin | 24 (13.2%) | 14 (13.1%) | 2 (11.8%) | 40 (13.1%) |  |
| Other cities | 38 (20.9%) | 18 (16.8%) | 4 (23.5%) | 60 (19.6%) |  |

**Table S2. Clinical Training Site Characteristics and Student Distribution**

| **Training Site** | **n** | **%** | **Mean Learning Experience Score** | **SD** | **95% CI** |
| --- | --- | --- | --- | --- | --- |
| **Governmental Hospitals** | 140 | 45.8 | 3.92 | 0.91 | [3.77, 4.07] |
| An-Najah National University Hospital | 52 | 17.0 | 3.89 | 0.88 | [3.65, 4.13] |
| Rafidia Hospital | 43 | 14.1 | 3.97 | 0.92 | [3.69, 4.25] |
| Thabet Thabet Hospital | 28 | 9.2 | 3.85 | 0.95 | [3.48, 4.22] |
| Palestine Medical Complex | 17 | 5.6 | 4.12 | 0.83 | [3.70, 4.54] |
| **Private Hospitals** | 117 | 38.2 | 3.59 | 1.06 | [3.40, 3.78] |
| Arab Specialized Hospital | 34 | 11.1 | 3.68 | 1.02 | [3.33, 4.03] |
| Al-Watani Hospital | 29 | 9.5 | 3.52 | 1.11 | [3.10, 3.94] |
| Private clinics (various) | 54 | 17.6 | 3.57 | 1.06 | [3.28, 3.86] |
| **UNRWA Clinics** | 49 | 16.0 | 3.87 | 0.88 | [3.62, 4.12] |

**Table S3. Clinical Ward Placements and Learning Experience Scores**

| **Clinical Ward** | **n** | **%** | **Mean Score** | **SD** | **Min** | **Max** | **95% CI** |
| --- | --- | --- | --- | --- | --- | --- | --- |
| Orthopedic | 12 | 3.9 | 4.92 | 0.15 | 4.65 | 5.00 | [4.83, 5.01] |
| Cardiac | 18 | 5.9 | 4.23 | 0.67 | 3.10 | 5.00 | [3.90, 4.56] |
| Intensive Care Unit | 48 | 15.7 | 4.15 | 0.82 | 2.20 | 5.00 | [3.91, 4.39] |
| Emergency | 22 | 7.2 | 3.98 | 0.91 | 2.30 | 5.00 | [3.58, 4.38] |
| Medical | 88 | 28.8 | 3.76 | 0.95 | 1.50 | 5.00 | [3.56, 3.96] |
| Surgical | 69 | 22.5 | 3.65 | 1.02 | 1.20 | 5.00 | [3.41, 3.89] |
| Maternity | 26 | 8.5 | 3.44 | 1.15 | 1.00 | 5.00 | [2.98, 3.90] |
| Pediatric | 23 | 7.5 | 2.97 | 1.32 | 1.00 | 5.00 | [2.40, 3.54] |

**Table S4. Correlation Matrix of CLES+T Dimensions and Learning Experiences**

| **Variable** | **1** | **2** | **3** | **4** | **5** | **6** |
| --- | --- | --- | --- | --- | --- | --- |
| 1. Pedagogical atmosphere | 1.000 |  |  |  |  |  |
| 2. Leadership style of ward manager | 0.672** | 1.000 |  |  |  |  |
| 3. Premises of care | 0.695** | 0.734** | 1.000 |  |  |  |
| 4. Supervisory relationship | 0.783** | 0.658** | 0.702** | 1.000 |  |  |
| 5. Role of nurse teacher | 0.724** | 0.689** | 0.715** | 0.748** | 1.000 |  |
| 6. Clinical learning experiences | 0.741** | 0.651** | 0.683** | 0.726** | 0.695** | 1.000 |

*Note: ** p < 0.001*

**Table S5. Post-Hoc Analysis Results for Clinical Ward Differences**

| **Ward Comparison** | **Mean Difference** | **SE** | **p-value** | **95% CI** |
| --- | --- | --- | --- | --- |
| Orthopedic vs Pediatric | 1.95* | 0.39 | < 0.001 | [1.04, 2.86] |
| Orthopedic vs Maternity | 1.48* | 0.36 | 0.001 | [0.63, 2.33] |
| Orthopedic vs Surgical | 1.27* | 0.32 | 0.002 | [0.52, 2.02] |
| Cardiac vs Pediatric | 1.26* | 0.31 | 0.002 | [0.54, 1.98] |
| ICU vs Pediatric | 1.18* | 0.25 | < 0.001 | [0.59, 1.77] |
| Emergency vs Pediatric | 1.01* | 0.29 | 0.012 | [0.34, 1.68] |
| Medical vs Pediatric | 0.79* | 0.23 | 0.018 | [0.25, 1.33] |
| Surgical vs Pediatric | 0.68* | 0.24 | 0.035 | [0.12, 1.24] |

*Note: * p < 0.05 (Tukey's HSD post-hoc test)*

**Table S6. Reliability Analysis of CLES+T Scale Dimensions**

| **CLES+T Dimension** | **Items** | **Cronbach's α** | **α if Item Deleted Range** | **Item-Total Correlation Range** |
| --- | --- | --- | --- | --- |
| Pedagogical atmosphere | 9 | 0.91 | 0.89 - 0.92 | 0.62 - 0.78 |
| Leadership style of ward manager | 4 | 0.89 | 0.84 - 0.91 | 0.71 - 0.84 |
| Premises of care | 4 | 0.87 | 0.82 - 0.89 | 0.68 - 0.79 |
| Supervisory relationship | 8 | 0.93 | 0.91 - 0.94 | 0.65 - 0.82 |
| Role of nurse teacher | 9 | 0.88 | 0.85 - 0.89 | 0.58 - 0.75 |
| **Total Scale** | **34** | **0.984** | **0.976 - 0.985** | **0.54 - 0.85** |

**Table S7. Qualitative Interview Participant Characteristics**

| **Participant** | **Gender** | **University Type** | **Clinical Placements Completed** | **Interview Duration (minutes)** |
| --- | --- | --- | --- | --- |
| P1 | Female | Private | 7 | 52 |
| P2 | Male | Private | 6 | 48 |
| P3 | Female | Public | 8 | 61 |
| P4 | Female | Private | 5 | 45 |
| P5 | Male | Governmental | 9 | 58 |
| P6 | Female | Public | 7 | 53 |
| P7 | Female | Private | 6 | 47 |
| P8 | Male | Private | 4 | 42 |
| P9 | Female | Private | 8 | 59 |
| P10 | Female | Public | 7 | 51 |
| P11 | Male | Private | 5 | 46 |
| P12 | Female | Governmental | 8 | 55 |
| P13 | Male | Public | 6 | 49 |
| P14 | Female | Private | 7 | 54 |

**Table S8. Qualitative Themes and Sub-themes with Supporting Quotes**

| **Main Theme** | **Sub-theme** | **Representative Quote** | **Participant** |
| --- | --- | --- | --- |
| **Perceptions of Clinical Experiences** | Theory-practice gap | "What we learn in theory is perfect, but reality is messy and unpredictable" | P3 |
|  | Real-world learning | "Touching real patients, seeing real conditions - that's when learning becomes meaningful" | P7 |
|  | Emotional impact | "Sometimes it's overwhelming, but it makes us grow as future nurses" | P12 |
| **Facilitators of Learning** | Instructor expertise | "Good instructors don't just teach, they inspire confidence in us" | P1 |
|  | Collaborative staff | "When nurses treat us as team members, not just students, we learn more" | P9 |
|  | Supportive environment | "Feeling safe to make mistakes and learn from them is crucial" | P14 |
| **Barriers to Learning** | Resource limitations | "Sometimes 10 students share one stethoscope - how can we practice properly?" | P5 |
|  | Movement restrictions | "Checkpoints make us late, stressed, and we miss important sessions" | P8 |
|  | Non-educational tasks | "We spend too much time on paperwork instead of patient care" | P11 |
| **Improvement Strategies** | Extended clinical time | "We need more hours in clinics to really understand nursing practice" | P2 |
|  | Diverse placements | "Seeing different hospitals would give us broader perspectives" | P6 |
|  | Better preparation | "More simulation before real patients would boost our confidence" | P10 |

**Table S9. Statistical Assumptions Testing Results**

| **Test** | **Assumption** | **Method** | **Result** | **Interpretation** |
| --- | --- | --- | --- | --- |
| ANOVA | Normality | Kolmogorov-Smirnov | p > 0.05 for all groups | Assumption met |
|  | Homogeneity of variance | Levene's test | F = 1.82, p = 0.164 | Assumption met |
|  | Independence | Study design | Random sampling within strata | Assumption met |
| Multiple Regression | Normality of residuals | Shapiro-Wilk | W = 0.993, p = 0.112 | Assumption met |
|  | Linearity | Scatterplots | Linear relationships observed | Assumption met |
|  | Homoscedasticity | Breusch-Pagan test | χ² = 14.3, p = 0.281 | Assumption met |
|  | Multicollinearity | VIF values | Range: 1.12 - 3.90 | Assumption met (VIF < 5) |
|  | Independence of errors | Durbin-Watson | DW = 1.89 | Assumption met (1.5 < DW < 2.5) |

**Figure S1. Distribution of CLES+T Scores by University Type**


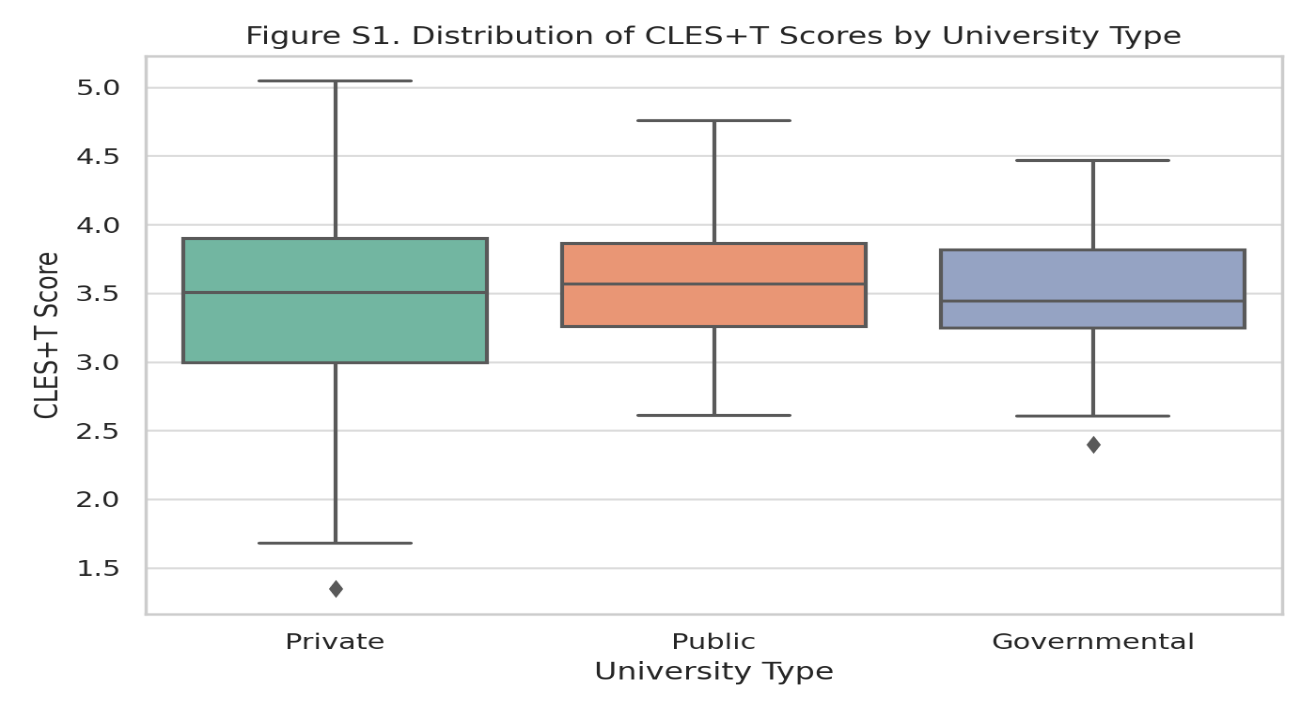


**Figure S2. Clinical Learning Experience Scores by Training Site**


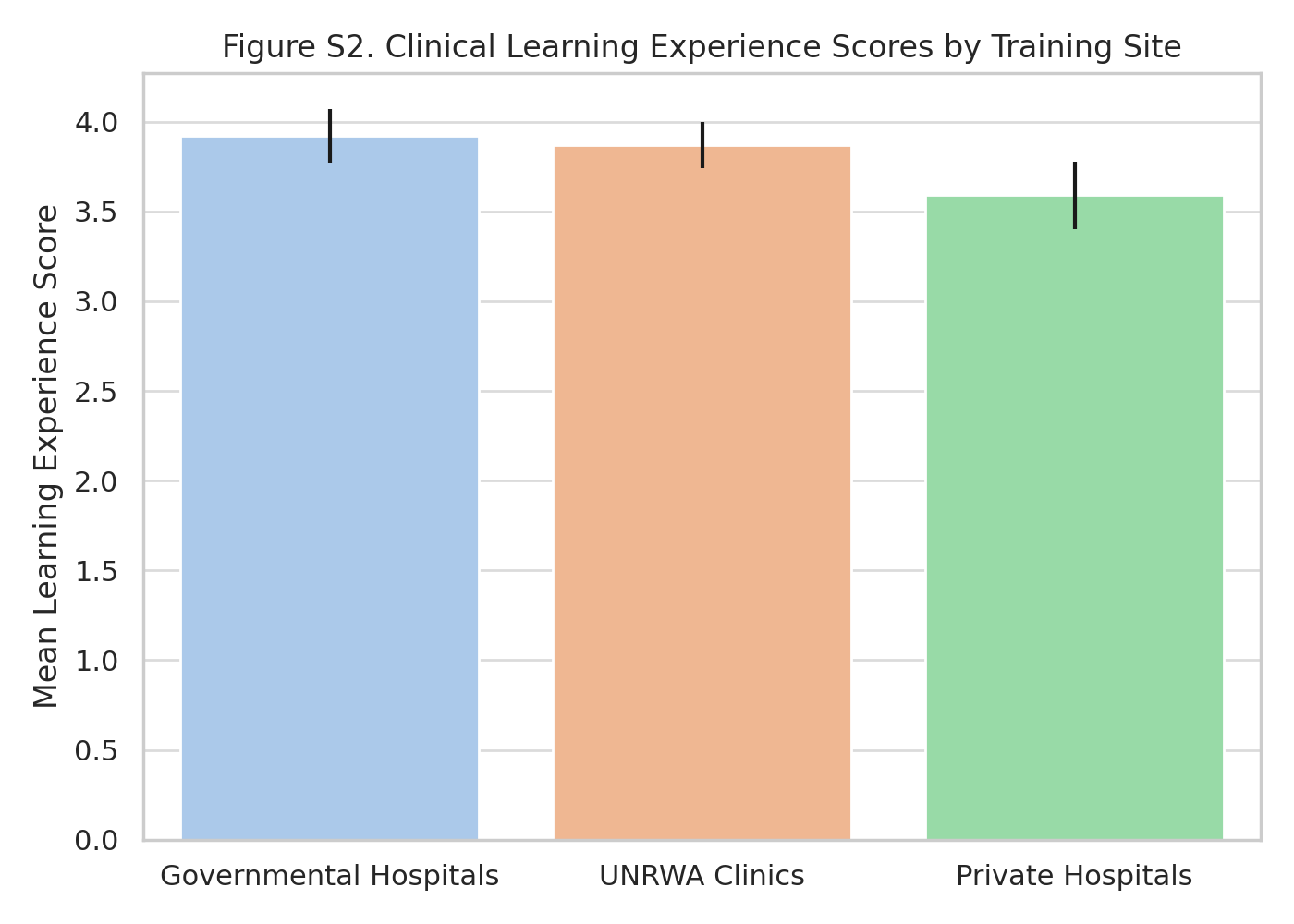


**Figure S3. Correlation Heatmap of CLES+T Dimensions**


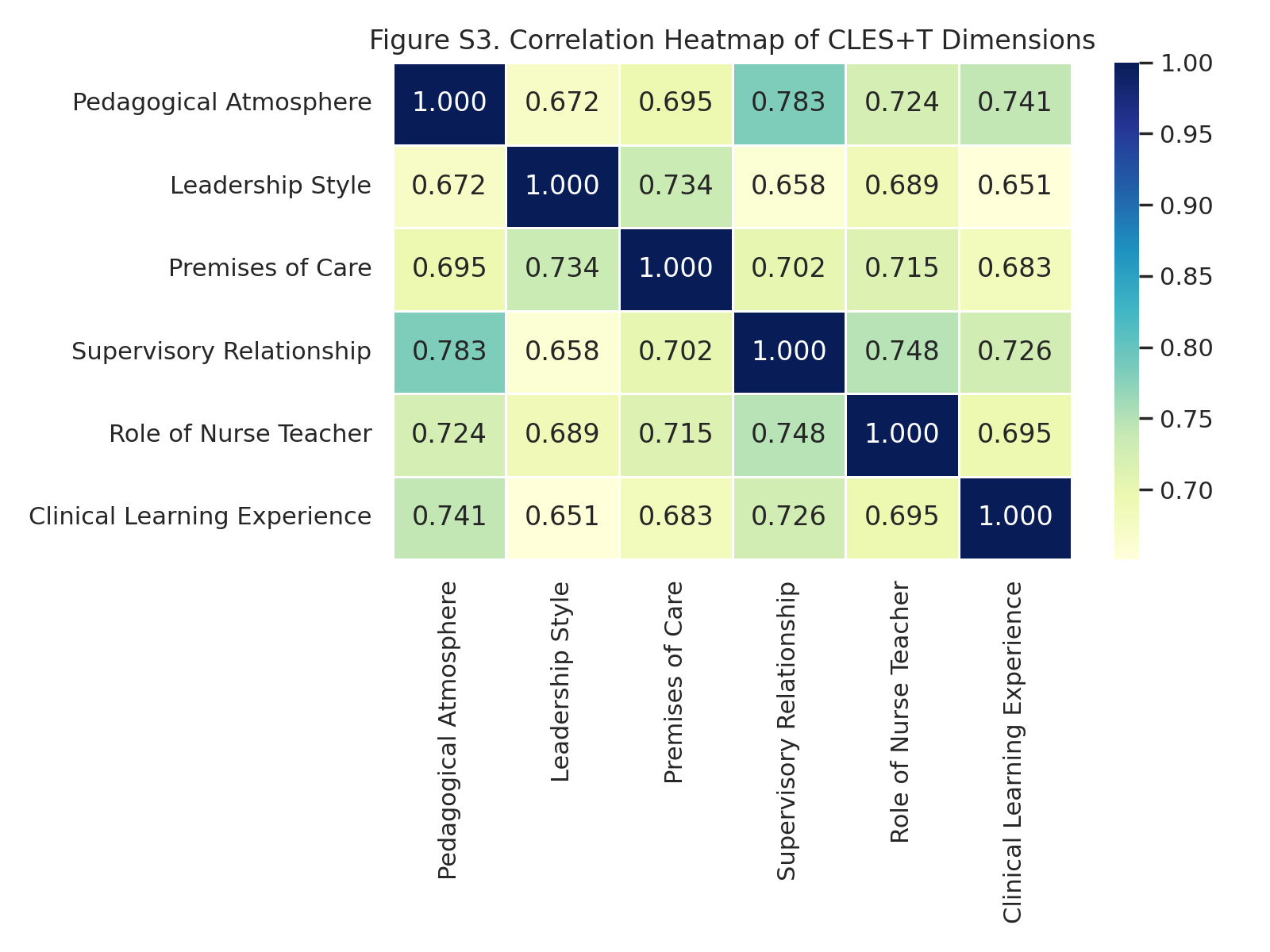


**Figure S4. Residual Plots for Multiple Regression Analysis**


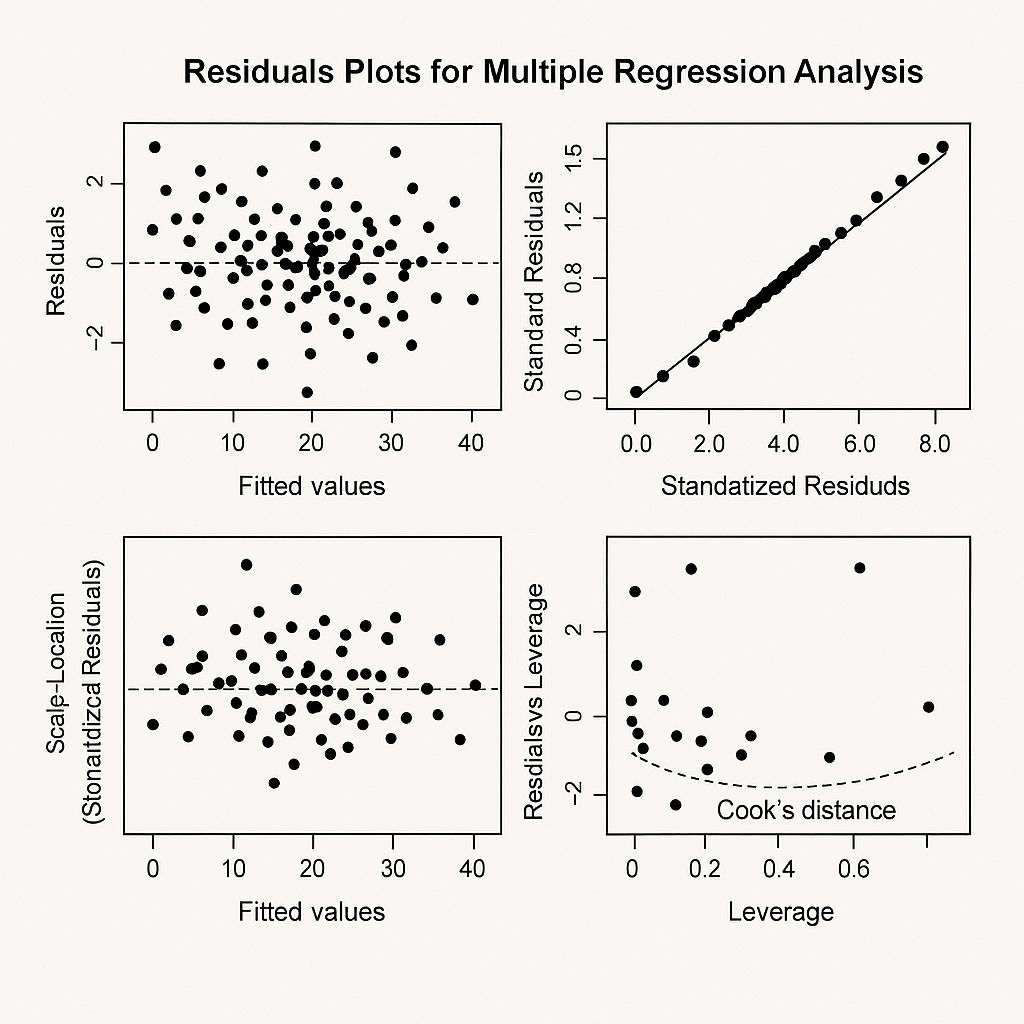


**Figure S5. Thematic Map of Qualitative Findings**


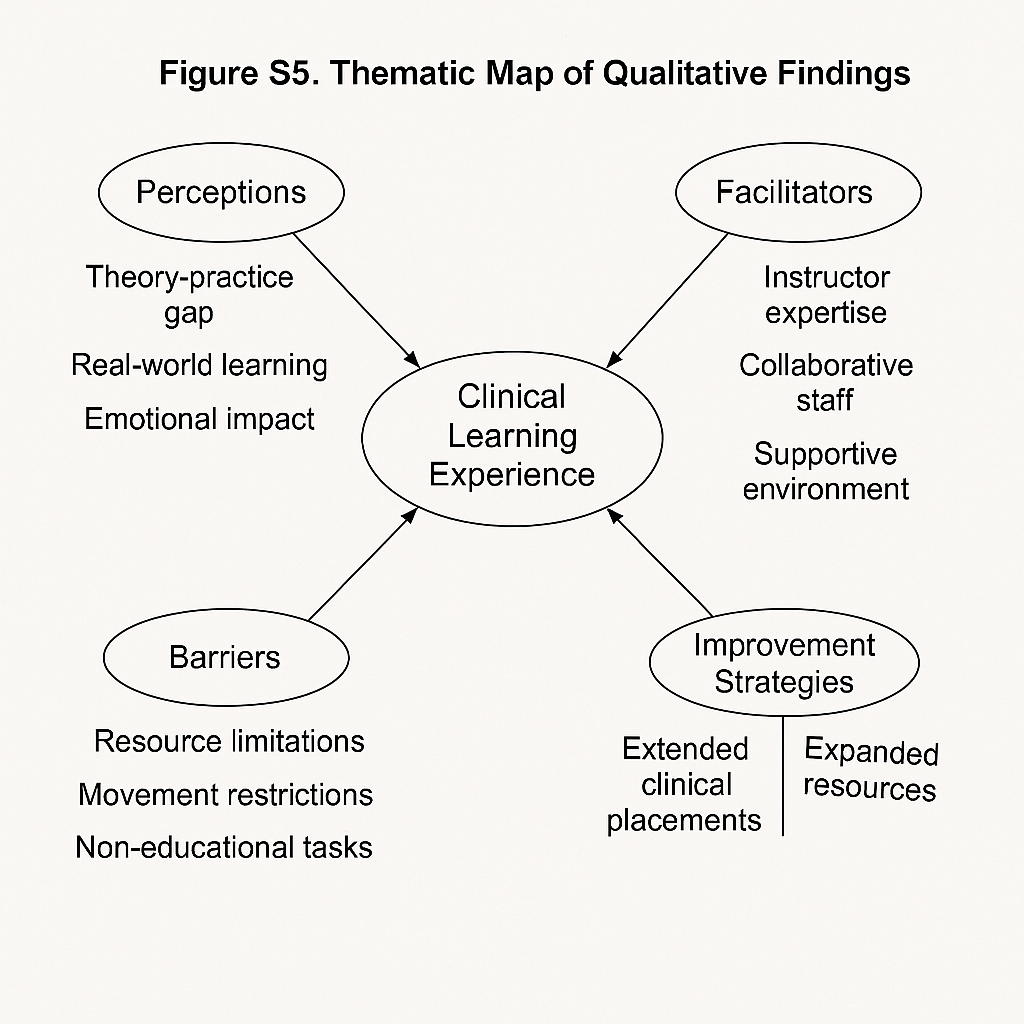


**Appendix A. Cultural Adaptation Process for CLES+T Scale**

The cultural adaptation of the CLES+T scale for the Palestinian context involved several systematic steps:

1. **Translation Review**: The existing Arabic version was reviewed by two bilingual nursing educators
2. **Cultural Adaptation**: Terms were modified to reflect Palestinian healthcare terminology
3. **Expert Panel Review**: Five experts evaluated content validity (CVI = 0.92)
4. **Pilot Testing**: Scale was tested with 30 students from each university type
5. **Final Validation**: Psychometric properties were confirmed with the full sample

**Key Terminological Adaptations:**

- "Nurse teacher" → "المدرس السريري" (Clinical instructor)
- "Ward" → "قسم" (Department)
- "Mentorship" → "التوجيه والإرشاد" (Guidance and counseling)

**Appendix B. Interview Guide Structure**

**Opening Questions:**

1. Tell me about your overall experience in clinical training
2. How would you describe the clinical learning environment in your placements?

**Main Questions:** 3. What factors make clinical learning effective for you? 4. What challenges do you face in clinical settings? 5. How do instructors and staff influence your learning? 6. What would improve your clinical learning experience?

**Closing Questions:** 7. How has clinical training shaped your view of nursing? 8. What advice would you give to improve clinical education?

**Appendix C. Missing Data Analysis**

| **Variable** | **Missing n (%)** | **Pattern** | **Handling Method** |
| --- | --- | --- | --- |
| Age | 2 (0.7%) | Random | Mean imputation |
| University type | 0 (0.0%) | None | No action needed |
| Clinical site | 3 (1.0%) | Random | Mode imputation |
| CLES+T items | 8 (2.6%) | Random | Listwise deletion |
| Learning experience | 4 (1.3%) | Random | Mean imputation |

**Little's MCAR Test**: χ² = 23.4, df = 28, p = 0.705 (Missing completely at random)
