# Supplementary material for: Clinical learning environments and experiences of nursing students in West Bank Universities: A mixed-methods study
Source: PLoS One. 2025 Aug 26;20(8):e0327506. doi: 10.1371/journal.pone.0327506 (PMC12380295; doi:10.1371/journal.pone.0327506)
Supplement: Attachment — Submitted filename: Response to Reviewers.pdf [file pone.0327506.s005.pdf]

Point-by-Point Response Table

Reviewer	Comment #	Item	Comment	Response	Location in Manuscript
**Editor**	1	Statistics Power	Explain why power=0.90 was used instead of 0.80.	Thank you. Added justification in Methods: "The use of power=0.90 (vs. conventional 0.80) was chosen to increase statistical power and reduce Type II error, given the exploratory nature of this study in a conflict-affected context where effect sizes might be more variable."	Methods, "Sampling Strategy and Power Analysis" (p. 4)
**Editor**	2	Eligibility Criteria	Specify age range and gender of participants.	Thank you. Added: "Eligibility criteria included... age range of 19–26 years, and both male and female students were included to ensure gender representation."	Methods, "Participants and Sampling" (p. 4-5)
**Editor**	3	PLOS ONE Style	Ensure compliance with formatting requirements.	Thank you. The manuscript now adheres to PLOS ONE's formatting guidelines, including file naming (e.g., "Revised_Manuscript.docx") and structure (e.g., headings, references).	Entire manuscript
**Editor**	4	Data Availability	Clarify restrictions or provide repository links.	Thank you. The Data Availability Statement now explicitly states: "Datasets are not publicly available due to ethical restrictions... but anonymized data may be made available upon request to the corresponding author (Dr. Ibrahim Aqtam) with IRB approval."	Data Availability Statement (p. 14)

**Editor**	5	Supplementary Captions	Add captions for supporting files.	Thank you. Added under "Supplementary Materials": **S1 File.** Checklist of Mixed Methods Elements; **S2 File.** Interview Guide; **S3 File.** Demographic Questionnaire; **S4 File.** Additional Statistical Tables.	Supplementary Materials (p.15)
**Reviewer 1**	1	Research Objectives	Explicitly state research questions/hypotheses and highlight novelty.	Thank you. Added in Introduction: Three research questions (e.g., CLE components' influence on learning) and novelty ("unprecedented insights into educational resilience in conflict zones").	Introduction (pp. 4–5)
**Reviewer 1**	2	Methodological Clarity	Clarify sampling strategy, sample size calculation, and quantitative/qualitative assumptions.	Thank you. Added: (1) Convenience sampling, stratified by university type; (2) G*Power analysis (n=306 exceeds thresholds); (3) Normality/multicollinearity checks; (4) Dual qualitative coding (κ=0.85).	Methods (pp. 4–6)
**Reviewer 1**	3	Limitations	Discuss self-report bias and selection bias.	Thank you. Added: "Self-report bias may have influenced responses... selection bias may exist due to underrepresentation of governmental university students (5.6%)."	Limitations (p. 12)
**Reviewer 1**	4	Ethical Considerations	Expand confidentiality, data security, and voluntariness.	Thank you. Added: "Data security measures included encrypted files, locked cabinets... Participants were explicitly informed of their right to withdraw without penalty."	Ethics Approval (p. 7)
**Reviewer 1**	5	Practical Implications	Provide concrete recommendations for stakeholders.	Thank you. Added actionable strategies for policy-makers (e.g., standardized protocols), educators (e.g., stress management training),	Practical Implications (pp. 12–13)

				and supervisors (e.g., structured orientation programs).	
**Reviewer 1**	6	Future Research	Suggest longitudinal/comparative studies.	Thank you. Added: "Future research should track outcomes longitudinally and compare CLEs in other conflict zones (e.g., Syria, Yemen)."	Future Research Directions (p. 13)
**Reviewer 2**	1	Introduction Enhancement	Cite suggested studies on stress/coping (Toqan et al., Ayed & Amoudi).	Thank you. Added citations to Toqan et al. (2022, 2023) and Ayed & Amoudi (2020) to contextualize stress management in clinical learning.	Introduction (p. 3, Refs 7–10)
**Reviewer 2**	2	Quantitative Reliability	Report Cronbach's alpha for CLES+T scale.	Thank you. Added: "Cronbach's alpha for CLES+T subscales: pedagogical atmosphere (α=0.91), supervisory relationship (α=0.93), etc."	Methods, "Instrument" (p. 5)
